# Herpes Simplex Virus (HSV)-Induced Erythema Multiforme: A Rare Case Report

**DOI:** 10.7759/cureus.62650

**Published:** 2024-06-18

**Authors:** Sourabh B Shinde, Vidya Lohe, Swapnil Mohod

**Affiliations:** 1 Oral Medicine and Radiology, Sharad Pawar Dental College and Hospital, Wardha, IND

**Keywords:** recurrent oral ulcer, oral mucosal ulcers, ‏acyclovir, recurrent erythema multiforme, hsv-1

## Abstract

A polymorphous recurrent eruption mostly composed of macules, bullae, papules, and target lesions, which are often distributed symmetrically and can spread to distant extremities, and oral mucosae are the features associated with erythema multiforme (EM). Herpes simplex virus (HSV) is a common condition that is associated with EM and manifests in late adulthood. It shows recurrence and is usually diagnosed clinically. Following is a case of HSV-associated EM. A 45-year-old patient visited the outpatient department with complaints of oral ulceration and associated pain and burning sensation. The patient also reported that similar ulcers were seen two months prior to her visit, which resolved on their own and the recurrence was seen two days prior to the visit. The recurrence occurred with more severity of pain and inflammation as compared to previous ulcers. The patient was kept on a combination therapy of antivirals, steroids, silymarin, and multivitamins for four visits with a tapering dose of steroids. Post-treatment, there was no recurrence till date and the patient is able to perform mastication as well as deglutition without any pain or burning sensation.

## Introduction

Erythema multiforme (EM) is a self-limiting condition of the skin with persistent, recurrent, and isolated lesions. It can be further subdivided into erythema minor, affecting only the skin, and erythema major with mucocutaneous involvement. Previously, it was considered similar to Steven-Johnsons syndrome (SJS) and toxic epidermal necrolysis (TEN). It is now established that EM is a different entity. With an annual incidence of 1%, its presence is seen most in adults who are younger than 40 years, with no racial association [1].

It can be associated with drugs or can be seen in cases of infection including the herpes simplex virus (HSV) [1,2]. The association is most common with HSV-1 but it can also occur with HSV-2 [1]. In cases of EM most associated with HSV infection, around 90% of cases of EM are due to HSV infection and the remaining 10% are drug-induced [1,2]. It is suspected that herpes simplex-associated erythema multiforme (HAEM) consists of an immune component that is directed to HSV antigen and expressed in the skin that contains the lesions. This is due to higher HSV-specific antibody response in HAEM compared to HSV patients with the infiltration of T-cells. These T-cells are found to be CD4+ T cells (CTL) and are principal responders to HSV antigens that show delayed hypersensitivity. This presentation is not seen in that of drug-induced EM where there is the presentation of CD8+ T cells [2].

EM usually is mild with no prodromal symptoms and of acute onset that usually starts with fever. Headache, cough, sore throat, malaise, lymphadenopathy, and polyarthralgia are the symptoms that can be seen within a week of the appearance of blisters. Lesions appear as "irregular red macules, papules and vesicles", which usually collapse to form plaques on the skin. A bull's eye appearance is seen due to crusting and blistering of the lesions in the center portion. The presentation of the oral mucosa is different than that of the skin; it contains erythematous macules followed by epithelial necrosis with a robust inflammatory halo [3].

Drug-induced erythema multiforme usually involves lips, oral mucosa, and bulbar conjunctiva with formations of erosions on lips and oral cavity due to rupture of bullae seen as the disease progresses. Even though the lesions show peculiar characteristics for each type of EM, they still can be very difficult to differentiate due to their overlapping features [4].

The diagnosis is easier as it mainly focuses on clinical lesions typically known as "target lesions" and a history or coexisting infection of HSV. It can contain skin lesions, oral lesions, or both. The confirmatory diagnosis is made usually after the detection of specific immunoglobulin M (IgM) or IgG antibodies, for which a test for serology for the identification of HSV-1 and HSV-2 should be done. According to some studies, HSV DNA has been detected in a significant number of patients with HSV-EM, ranging between 36% to 81%, by polymerase chain reaction amplification [5].

## Case presentation

**Figure 1:**
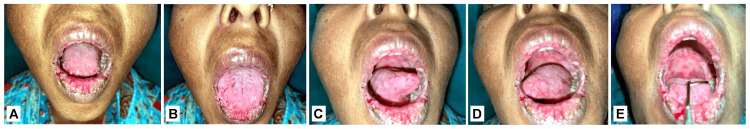
Crustations, ulcerations, and sloughing on the different mucosae of the oral cavity: A: crustations on the lower lip; B: sloughing and cracks on the dorsal surface of the tongue; C: ulceration and sloughing on right lateral border of the tongue; D: ulceration and sloughing on left lateral border of the tongue; E: ulcerations on the soft palate and uvula extending downward to the oropharynx

A female patient aged 45 years visited the outpatient department with the complaint of “recurrent oral ulcerations and pain with a burning sensation on the oral mucosae while eating and swallowing food or water since the last 15 days.” The patient was symptomless 15-20 days back. The patient gave a history of visits to a gynecologist regarding problems with the menstrual cycle but did not disclose the details of the visit. There she was advised of some medications. A day after the administration of medications, the patient started noticing crops of vesicles, which broke into the multiple ulcers on site, which were painful. Initially, the ulcers were smaller in size and the pain was of lesser intensity and they gradually increased in size and intensity at the time of examination. The Visual Analogue Score (VAS) was 7 on a scale of 10 (0 being no pain and 10 being unbearable pain). The patient had difficulty in mouth opening as she had severe dehydration, which led to cracks in the mucosa of the upper and lower lips, which further led to bleeding while mouth opening as it stretched the mucosae. The bleeding was also observed from ulcers on palpation. The patient also complained of burning micturition and indigestion. On extra oral examination, the face was symmetrical as there were no swellings or ulcerations present on the face. Lips showed crustated ulcerations along with bleeding points. The ulcers bled vaguely on the application of tension to the mucosae. There was submandibular lymphadenopathy present bilaterally, where a single node was palpable, which was soft, mobile, and non-tender. On intra-oral examination, there were multiple round ulcerations seen bilaterally on the lateral border of the tongue, dorsal surface of the tongue, buccal mucosa, hard palate, and soft palate and they extended further downwards to the uvula and oropharynx. The ulcers were covered with necrotic slough. The dorsal surface of the tongue also showed cracking in the tongue mucosa (Figure 1). With the above history and clinical features, herpes simplex virus-associated erythema multiforme was given as a provisional diagnosis. The Steven-Johnson syndrome was advised as a differential diagnosis.

**Figure 2:**
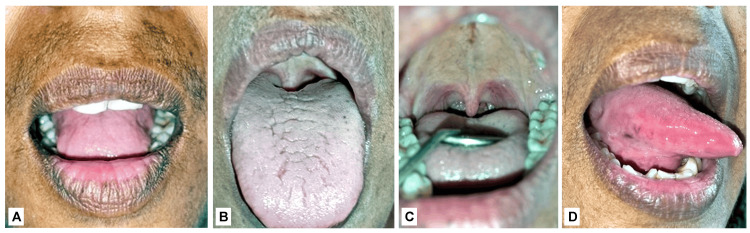
A-D: Complete healing of the ulcer can be seen.

The patient was advised to tab. prednisolone 20mg 12 hourly for five days, tab. acyclovir 400mg 12 hourly for five days, tab. pantoprazole + domperidone 12 hourly for five days, and local application of triamcinolone acetonide cream on the ulcers six hourly for five days. The patient was recalled after five days. On the second visit, the patient reported 50% relief from all the symptoms with a VAS score of 5 on a scale of 10 (0 being no pain and 10 being unbearable pain). The patient was now able to eat mild and bland food but still was sensitive to spicy food, and able to drink water. The patient also experienced 60% relief from burning micturition. The ulcers recovered by 60% and bleeding points were reduced by 60-70%. The patient was advised tab. prednisolone 10mg 12 hourly for seven days, tab. acyclovir 400mg for seven days, tab. silymarin + L- glutathione + N-acetyl-L-cysteine + L-carnitine + L-ornithine + coline combination 12 hourly, tab. B-complex + B12 12 hourly, and tab. pantoprazole + domperidone 12 hourly for a further seven days and local application of triamcinolone acetonide cream on the ulcers six hourly was also continued for further seven days. The patient was recalled after seven days. On the third visit, the patient reported 80% relief from all the symptoms with a VAS score of 2 on a scale of 10 and complete recovery from burning micturition. The ulcers were completely healed and bleeding points also reduced by 90%. Prednisolone dosage was reduced to 5mg and the patient was advised to gargle 24 hourly for seven days. Acyclovir was discontinued as the ulcers were completely healed. The pantoprazole and domperidone combination was also discontinued. All the remaining medications were again continued for seven days. The patient was recalled after seven days. On the fourth visit, the patient reported a complete recovery from all the symptoms with a VAS score of 0 on a scale of 10. On clinical examination the ulcers showed complete healing and so was the case with sloughing. The patient also reported a complete relief from dehydration as no crustations or bleeding points observed on the lips (Figure 2).

## Discussion

Epidermal cell death, also termed satellite cell necrosis, is the characteristic histopathological change of the EM minor that mimics the apoptotic cell death. Among the substances that induce apoptosis, a pore-making granule from natural killer cells named perforin has been suggested. The changed expression of apoptotic regulatory proteins is another mechanism that can be related to EM minor. The Bcl-2 protein and its intense expression under inflammatory cells in EM minor play a role in this protein maintaining the infiltrate in the submucosa. Fas antigen and its altered expression throughout the epithelium compared with the inflammatory cell infiltrate has been reported in EM minor. On the other hand, EM major is distinguished by an involvement of multiple mucous membranes, which can be termed as the more severe form of the EM minor. In addition to the oral cavity, the laryngeal and oesophageal mucosae can be affected [5]. In this particular case, multiple mucosae were affected.

Table 1 shows the subtypes of erythema multiforme.

**Table 1:** Subtypes of erythema multiforme with their clinical features

Subtypes of erythema multiforme	Clinical features
Erythema multiforme minor	Raised typical or atypical target lesions with minimal involvement of the mucus membrane, with presence noted on a single site. Commonly involving oral mucosa with erythema, erosions, and ulcers. Less than 10% of the surface area of the body is involved.
Erythema multiforme major	Commonly involving two or more sites (more typical oral mucosa involvement) with cutaneous lesions. Less than 10% of the surface area of the body is involved. The distribution is seen in symmetry with target lesions being raised, typical/atypical, or both. Lesions are usually severe.
Steven–Johnson syndrome	It differs from erythema multiforme major, distinguished by the presence of the type and location of the lesions with systemic symptoms. Less than 10% of the surface area of the body is involved. Rather than classic target lesions, atypical flat target lesions and macules are characteristics of this syndrome. Commonly seen with flu-like symptoms.
Overlapping Steven–Johnson syndrome and toxic epidermal necrolysis	Atypical, flat targets are present rather than typical targets Up to 10–30% of the surface area of the body is involved. Prodromal flu-like systemic symptoms are common.
Toxic epidermal necrolysis	In the presence of spots. More than 30% of the surface area of the body is involved and associated with epidermal detachment, which is a characteristic feature. Another characteristic feature is atypical, flat targets and widespread purpuric macules with absence of spots. Less than 10% surface area of the body is involved. Commonly associated with epidermal detachment with large epidermal sheets. There is an absence of macules or target lesions.

The symptoms sometimes overlap with other acute oral ulcerative conditions, most commonly in childhood. Conditions like hand foot and mouth disease usually overlap symptoms with HSV. Recurrence is seen as herpes labialis on the vermillion border of the lip and it is experienced by almost 40% of the patients. Intra-oral ulcerations are limited to the attached gingiva, which begin as small, painless, multiple ulcerations that heal within 10 days. Non-keratinized mucosa can be involved in immunocompromised patients [6]. The recurrence is also documented and in the present case, the patient gave a history of similar ulcers two months earlier correlating the recurrence. In the case of erosive oral lichen planus, ulcers are also present, causing burning sensations, but the presence of striations is ruled out [7]. The Zosteriform herpes simplex infection can also lead to ulceration, pain, and burning, but unilaterality is the hallmark of this condition [8]. The graft versus host disease, especially the skin grafts, usually resembles herpes-associated EM. It is because of its association with non-replicative herpes simplex infection, which further shows its association with viral DNA fragmentation that is present inside the infected CD34+ cells. They further transport the fragments to the skin [9]. This was not the case of graft versus host reaction.

The diagnostic part of the EM is mainly based on thorough history taking and clinical presentation of the lesions, whereas histopathology and laboratory investigations are not specific. An increased positive rate of IgG antibodies of HSV higher than that of IgM amongst healthy people was found by Song et al. (cited in [10]). It might be due to a period of interval between the present asymptomatic infection and the detection of antibodies [10]. This case was also diagnosed clinically. The treatment plan should be adhered to the symptoms, i.e. first the etiologic cause of the symptoms should be identified. If the cause is drug-induced, an immediate stoppage of that drug should be done and drugs with similar chemical formulation should be avoided. In HSV-induced EM, acyclovir is the drug of choice while macrolides like azithromycin are the drugs of choice in mycoplasma-induced EM. Systemically given corticosteroids are used for the management of acute outbreaks. If corticosteroids are not administered, the patient will recover from minor outbreaks at a slower rate, but it will not be sufficient for major outbreaks [11].

In the discussed case, as the etiology was viral and history suggested recurrence of the lesions after two months, the patient was administered tab. acyclovir 400 mg two times a day. In addition to that, steroids were administered on a tapering dose (20 mg twice daily, followed by once daily, followed by gargles). Local application of triamcinolone acetonide was also advised to reduce the local inflammation as the patient had difficulty in eating and swallowing. The patient was also advised tab. pantoprazole as the patient experienced indigestion and to avoid gastric inflammation.

## Conclusions

HSV is one of the most common viral infections and is seen primarily in children and young adults. Its recurrence, though not common, shows moderate to severe clinical features with possible recurrence. Hence a precise and updated knowledge is necessary and diagnosis through clinical investigation plays a very important role as the condition mimics many other diseases. The treatment and post-treatment follow-up are equally important to minimize the chances of recurrence.
